# Cognitively-plausible reinforcement learning in epidemiological agent-based simulations

**DOI:** 10.3389/fepid.2025.1563731

**Published:** 2025-07-28

**Authors:** Konstantinos Mitsopoulos, Lawrence Baker, Christian Lebiere, Peter Pirolli, Mark Orr, Raffaele Vardavas

**Affiliations:** ^1^ Florida Institute for Human and Machine Cognition, Pensacola, FL, United States; ^2^ RAND Corporation, Boston, MA, United States; ^3^ Department of Psychology, Carnegie Mellon University, Pittsburgh, PA, United States; ^4^ RAND Corporation, Santa Monica, CA, United States

**Keywords:** infectious disease modeling, reinforcement learning, ACT-R, agent-based modeling, cognitive modeling

## Abstract

**Introduction:**

Human behavior shapes the transmission of infectious diseases and determines the effectiveness of public health measures designed to mitigate transmission. To accurately reflect these dynamics, epidemiological simulation models should endogenously account for both disease transmission and behavioral dynamics. Traditional agent-based models (ABMs) often rely on simplified rules to represent behavior, limiting their ability to capture complex decision-making processes and cognitive dynamics.

**Methods:**

Reinforcement Learning (RL) provides a framework for modeling how agents adapt their behavior based on experience and feedback. However, implementing cognitively plausible RL in ABMs is challenging due to high-dimensional state spaces. We propose a novel framework based on Adaptive Control of Thought-Rational (ACT-R) principles and Instance-Based Learning (IBL), which enables agents to dynamically adapt their behavior using nonparametric RL without requiring extensive training on large datasets.

**Results:**

To demonstrate this framework, we model mask-wearing behavior during the COVID-19 pandemic, highlighting how individual decisions and social network structures influence disease transmission. Simulations reveal that local social cues drive tightly clustered masking behavior (slope = 0.54, Pearson *r* = 0.76), while reliance on global cues alone produces weakly disassortative patterns (slope = 0.05, Pearson *r* = 0.09), underscoring the role of local information in coordinating public health compliance.

**Discussion:**

Our results show that this framework provides a scalable and cognitively interpretable approach to integrating adaptive decision-making into epidemiological simulations, offering actionable insights for public health policy.

## 1 Introduction

Disease transmission is influenced by both biological factors and human behavior. Public health interventions–such as limiting social contact, promoting vaccination, and encouraging mask-wearing–play a critical role in controlling its transmission. The COVID-19 pandemic, in particular, revealed the challenges of understanding how populations respond to these interventions and their effectiveness in mitigating transmission (1, 2). Although researchers have created models to predict disease transmission and evaluate the effectiveness of these interventions (3), there is a significant gap in understanding how adaptive behaviors interact with social network structures and influence disease epidemiology (4, 5).

Agent-based models are used to simulate individual characteristics and interactions within populations, offering a computational approach to studying emerging behaviors and epidemiological dynamics. The COVID-19 pandemic demonstrated the importance of incorporating adaptive decision-making and changing preferences for social distancing and vaccination, as these decisions significantly impact disease transmission and the effectiveness of public health interventions (3). However, many ABMs rely on simple, rule-based representations of behavior that fail to capture the complexity of human decision-making and cognition.

Reinforcement Learning (RL) is a computational framework inspired by behavioral psychology, particularly operant conditioning, that models how agents learn to make decisions by interacting with an environment to maximize utility through experience. In the context of human decision making, RL provides a framework to understand and simulate how humans learn from the consequences of their actions, adapt their behavior over time, and make choices under uncertainty. RL is particularly suited for decision-making in dynamic environments, as it can represent mechanisms such as risk assessment, habit formation, and goal-directed behavior. Despite these advantages, incorporating cognitively plausible RL into agent-based simulations is challenging. The high-dimensional state spaces typical in ABMs require function approximators, such as neural networks, to estimate expected utilities. However, these models are often not interpretable, require training on large datasets, and are computationally expensive.

To address these challenges, we propose a framework based on Adaptive Control of Thought—Rational (ACT-R) principles and Instance-based Learning (IBL). ACT-R provides a cognitively grounded architecture for modeling human cognitive processes, while IBL offers a non-parametric approach for learning and decision-making. Our framework avoids the need for explicit training phase and instead, dynamically adapts to new information by leveraging past experiences stored in the architecture’s memory. This enables agents to make decisions that are both adaptive and cognitively interpretable, aligning with human-like behavior.

We demonstrate the potential of this framework by applying it to mask-wearing behavior during the COVID-19 pandemic. Mask-wearing is an ideal intervention in which to study human behavior, because it involves frequent individual decisions that can adapt to changing circumstances. In contrast, decisions on lockdowns are made collectively for large groups and vaccination decisions usually occur annually. The model captures how individual decisions-shaped by personal risk tolerance, peer conformity, and discomfort-interact with social network structures to impact population-level infection outcomes. Our experiments show that this approach offers a scalable, flexible, and interpretable method for integrating data-driven cognitive modeling into epidemiological simulations, which can support public health policy-making.

The remainder of this paper is organized as follows. In Section 2, we review background literature and related work on epidemiological modeling, reinforcement learning, and cognitive architectures. Section 3 presents the theoretical foundations of our framework, outlining its statistical learning principles and cognitive mechanisms. In Section 4, we apply the framework to a case study on mask-wearing behavior during the COVID-19 pandemic. Section 5 reports simulation results examining how behavioral adaptation and network structure shape infection dynamics. Section 6 discusses the broader implications, advantages, and potential extensions of the framework. Finally, Section 7 concludes with limitations and future research directions.

## 2 Background and related work

Computational epidemiology combines multiple disciplines to study disease transmission and evaluate public health interventions (3). Effective policy analysis requires models that integrate causal epidemiological and behavioral theories with empirical data (6). Disease transmission in the real world involves complex behavioral dynamics influenced by demographics and the social norms (7). To address these requirements, there is a need to integrate endogenous behavior into epidemiological models of disease transmission (8–10). While such integrated approaches have existed for over a decade (11–15), the COVID-19 pandemic has resulted in renewed interest, particularly in modeling how compliance with interventions varies over time and its impact on disease epidemiology (16). Many epidemiological simulations use population-based models (PBMs), relying on differential equations to represent disease transmission (17). While PBMs can incorporate some population differences, they cannot capture individual behaviors or complex social networks. When combined with behavioral models, PBMs adjust disease transmission rates at the population or group level, rather than modeling how individuals adapt (18).

Sufficiently detailed behavioral simulations require a framework where individuals interact across complex social networks and make autonomous decisions as agents (19, 20). This has prompted the development of sophisticated models with deliberative agents, where variability in behaviors and decisions can emerge due to differences in individual epidemiological histories instead of only by aggregate-level group membership. Agent-based models have become essential in computational epidemiology to overcome the limitations of population-based models (21–24). However, Agent-based models typically use predefined rules to govern agent interactions and simulate resulting behaviors. This approach may not capture the emergence of complex and adaptable behaviors.

Reinforcement Learning (RL) provides a computational framework for understanding how agents learn to make decisions by trial and error to maximize rewards and minimize punishments (25). Its relevance to human behavior and cognition emerged with findings that RL algorithms mirror the activity of dopamine neurons, which encode prediction errors to guide learning and decision-making (26). These insights have been extended to explain the role of the basal ganglia and dopaminergic systems in motor control, habit formation, and reward-driven behavior (27, 28). By integrating neural mechanisms, RL approaches provide a framework for modeling higher-level cognitive functions such as planning, goal-directed behavior, cognitive control, and even simulating the interactions between the prefrontal cortex and basal ganglia (29, 30). Hierarchical RL approaches have further clarified how humans organize actions into structured sequences to achieve complex goals (31). Additionally, Bayesian extensions of RL have provided a framework for understanding adaptive and maladaptive behaviors, such as learned helplessness and the ability to infer others’ goals through theory of mind (32, 33).

RL approaches to modeling human behavior are typically applied to constrained state and action spaces, as these tasks are often designed to test specific aspects of cognition and are simpler in nature. However, agent-based simulations often involve large, non-enumerable state spaces, posing significant challenges for traditional RL methods. To address these challenges, value or policy functions are often approximated using parametric models such as neural networks, enabling Deep RL to solve high-dimensional tasks like Atari games (34, 35).

In computational epidemiology, Deep RL has been leveraged for various applications. For instance, (36) developed a deep learning framework using recurrent and convolutional neural networks to predict epidemiological conditions, such as patient counts and activity levels, in time-series data, outperforming traditional autoregressive models. Other studies have demonstrated the ability of Deep RL to learn effective mitigation policies under complex epidemiological conditions, across large state and action spaces (37, 38). Bushaj et al. (39) developed a Simulation-Deep Reinforcement Learning (SiRL) framework which can suggest optimal interventions based on specific epidemic situations and compare different vaccination strategies.

Beyond epidemiology, Deep RL has also been used along with agent-based models to study social phenomena. For example, (40) investigated the self-organizing dynamics of social segregation, revealing how reward structures influence segregation patterns and demographic distributions. Jäger (41, 42) proposed neural networks as replacements for manually defined behavioral rules in ABMs. Additionally, decision trees and random forests have been explored for behavior modeling in ABMs. However, these approaches face limitations, such as difficulties in ensuring realistic decision-making when agents lack critical information or when training environments differ significantly from application settings, often requiring iterative retraining to address these gaps effectively.

As (43) noted, Deep RL methods rely on incremental parameter adjustment through gradient descent. While effective, this process requires small updates to preserve generalization and avoid catastrophic interference, leading to slow learning (44, 45). Furthermore, the weak inductive bias of neural networks allows them to model a broad range of patterns but makes them highly data-intensive and sample-inefficient (46). These limitations result in Deep RL methods demanding orders of magnitude more training data than humans for similar tasks (47), making them less analogous to human learning and behavior.

Cognitive architectures provide a framework not only for modeling behavior but also for capturing the underlying cognitive processes and computational stages that drive decision-making. ACT-R is a cognitive architecture that integrates modules for memory, perception, and action to simulate human cognition (48). ACT-R has been used to model phenomena such as learning, fatigue, and goal-directed decision-making. Building on ACT-R principles, Cognitive Instance-Based Learning [CogIBL; (49)] enables non-parametric, instance-based function approximation, offering a cognitively interpretable alternative to neural network-based approaches. CogIBL has been used to model various aspects of human behavior across a range of domains such as competitive/cooperative games (50–53), cybersecurity (54, 55), and automated malware/intrusion detection systems (56).

The framework was investigated independently by Blundell et al. (57) and referred to as Episodic RL and was used to alleviate the issues associated with the parametric form of Deep RL. It was further extended to accommodate learned representations from neural networks (58). Related to our work is the concept of Psychologically Valid Agents (PVAs; (59–61)), which is based on computational agents implemented within the ACT-R architecture to simulate and analyze human behaviors in epidemiological settings. PVAs incorporate heterogeneous input drivers, such as media exposure and psychological traits, to model behavior dynamics. However, these approaches have primarily focused on regional dynamics rather than individual decision-making in large-scale social networks. Similarly, (62) developed an ACT-R-based model to simulate vaccination decisions influenced by personal and social network experiences, but their approach did not leverage the estimation capabilities and utility-based learning of ACT-R.

## 3 Cognitive framework

To address the aforementioned limitations, we build on this prior work to develop a computational framework that combines non-parametric machine learning, grounded in a cognitive architecture, with agent-based simulations to enable real-time, cognitively plausible decision-making. The machine learning foundation allows the agents for statistical inference for data-driven decision-making, instead of manually predefined rules. The architecture’s non-parametric, instance-based properties allow learning without distinct training and deployment phases, making the framework both sample-efficient and adaptive. Finally, the cognitive constraints provide interpretability and links behavior to cognitive and psychological theories. In this section, we describe the statistical learning foundations of the framework, the architecture and the benefits of the approach.

### 3.1 ACT-R theory summary

ACT-R is a cognitive theory that models decision-making as a production system operating over a declarative memory. The architecture assumes that cognition is shaped to perform optimally given the statistical structure of the environment, and emphasizes activation-based processes for relating the production system to the declarative memory. Different experiences in declarative memory have different levels of activation which determine their rates and probabilities of being processed by the production rules. These mechanisms allow agents to make decisions by retrieving information that is most relevant to the current situation. According to ACT-R theory, knowledge is divided into two distinct types:

• *Declarative knowledge*, which is stored in memory as structured units called *chunks*. These chunks represent factual or experiential knowledge that consists of: the input situation x consist of contextual features $x_{i}$ (e.g., local and global infection rates), the action a taken in that situation (e.g., whether to wear a mask), and the utility value that resulted from that decision.
• *Procedural knowledge*, which is encoded as production rules—symbolic if-then rules that govern behavior. Production rules control the flow of cognition by triggering actions or subgoals when specific conditions are met, and their utilities are updated over time through reinforcement-like learning mechanisms. This procedural component supports skill acquisition, strategic planning, and the execution of multi-step cognitive operations [as employed in (63–65)].

In this work, we focus exclusively on declarative knowledge, as we do not aim to model skill learning or goal-oriented behavioral sequences that require procedural knowledge. Instead, we rely on declarative mechanisms to estimate the utility of actions based on past experiences.

### 3.2 Statistical learning foundations

The core decision making component for each agent in our simulations is based on the CogIBL which is a cognitive framework implemented within the constraints of ACT-R principles. Although developed independent of Statistical Learning theory (66) and with utility-based learning in mind, CogIBL fundamentally employs the same principles as Instance-Based Learning [IBL; (67)], but adapts them to provide cognitively interpretable mechanisms. IBL is a family of Machine Learning algorithms that approximate functions based on comparisons between new problem instances with similar instances previously seen and stored in a memory module. This is in contrast to other methods such as neural networks that create abstract representations from specific instances. Specifically, CogIBL is a *linear smoother* (68, 69) which is a non-parametric^1^ instance-based learning function approximator. Therefore, CogIBL can implement various types of learning algorithms. These include Supervised Learning (SL), with applications in regression and classification, and RL, which facilitates utility-based learning for habitual behavior and with additional modules (e.g., goal buffers) it can support goal-driven behavior. Below, we outline the general statistical learning capabilities of CogIBL, starting with SL as this provides the regression mechanism which enables the utility function approximation in the RL case.

#### 3.2.1 Supervised learning capabilities

**Figure 1:**
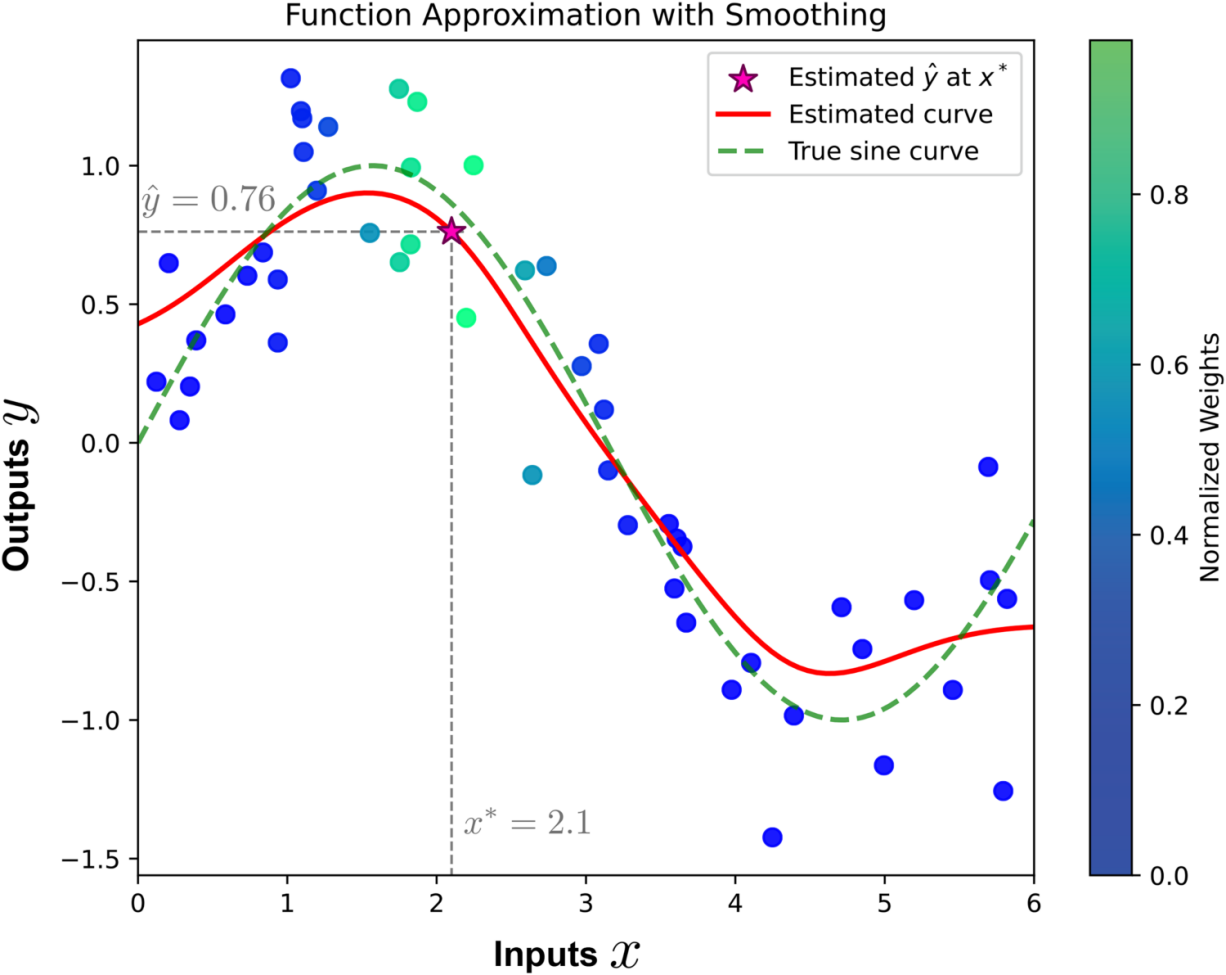
Illustration of function approximation using smoothing. The red curve represents the estimated function, while the green dashed curve shows the true sine function. The value $\hat{y}$ is estimated at the new input $x^{*}=2.1$ using a weighted average of observed outputs, where the weights are determined by the similarity between $x^{*}$ and the observed inputs. The point $\left(x^{*},\hat{y}\right)$ is indicated by the star symbol. The color bar indicates the normalized weights, with higher weights assigned to inputs closer to $x^{*}$.

The premise of SL is to learn a function that maps input data to corresponding outputs, based on provided examples of input-output pairs. Given samples $(x_{i},y_{i}),\;i=1,\ldots ,N$, where $x_{i}=(x_{i}^{1},x_{i}^{2},\ldots ,x_{i}^{D})$ is a D-dimensional vector of features with $x_{i}^{j}\in R$ for j=1,…,D, a linear smoother is an estimator for the underlying *regression* function f(x) at an arbitrary point $x_{0}$, expressed as:

(1) $$\hat{\;f}(x_{0})=\sum_{\;j=1}^{N}w(x_{0},x_{j})\cdot y_{j},$$

where $w(x_{0},x_{j})\in R$ are weights determined based on the similarity function w between the query point $x_{0}$ and each data point $x_{j}$ in the dataset, and $y_{j}$ represents the corresponding output. It is important to note that the estimator in Equation 1 directly minimizes the mean squared error between the predicted values $\hat{\;f}$ and true values y, as proven in Statistical Decision Theory (70, 71). This is in contrast to parametric approaches that require parameter estimation by minimizing the mean squared error. Figure 1 illustrates a one-dimensional regression example. To estimate the value $\hat{y}$ of the underlying unknown function for a new input $x^{*}$, the smoother computes a weighted average of the observed outputs. The weights are determined by the similarity between the new input and the observed inputs, with higher similarity resulting in greater weights.

For *classification* tasks, the target output $y_{j}$ is a discrete class label. In this case, the linear smoother estimates the probability of each class c at $x_{0}$ by aggregating the contributions of neighboring data points (Equation 2):

(2) $$\hat{P}(c|x_{0})=\sum_{\;j=1}^{n}w(x_{0},x_{j})\cdot I(y_{j}=c),$$

where $I(y_{j}=c)$ is an indicator function that equals 1 if $y_{j}$ belongs to class c, and 0 otherwise. The predicted class is then determined as the one with the highest estimated probability (Equation 3):

(3) $$\hat{y}=\arg\;\max_{c}\hat{P}(c|x_{0}).$$

This formulation allows linear smoothers to be applied for both regression and classification tasks. Time dependencies can be introduced into the framework either by adapting the similarity function $w(x_{0},x_{j})$ to account for temporal proximity or by incorporating an additional parametric term, such as a weighted sum of lagged values, creating a semi-parametric model. This modification enables the linear smoother to perform autoregressive computations modeling explicitly temporal dynamics. Moreover, the framework can be extended to handle non-linear relationships by allowing the weights to depend on both inputs and outputs, making the smoother non-linear with respect to the outputs (unlike the standard case where weights depend only on inputs and the smoother remains linear).

#### 3.2.2 Reinforcement learning capabilities

RL focuses on optimizing an agent’s sequential decision-making by maximizing cumulative rewards obtained through interaction with an environment. We consider the standard RL setting, where an agent interacts with an environment E over discrete time steps to complete a task. At each time step t, the agent observes the state $s_{t}$ of the environment and selects an action $a_{t}$ from a set of possible actions A, following its policy π. The policy π is a decision-making function that maps states $s_{t}$ to actions $a_{t}$. After taking the action, the agent transitions to the next state $s_{t+1}$ and receives a scalar reward $r_{t}$. This process continues until a terminal state is reached, after which the environment resets.

The goal of the agent is to maximize the expected return, defined as the total accumulated reward over time $R_{t}=\sum_{k=0}^{\infty }\gamma^{k}r_{t+k}$, where γ∈(0,1] is a discount factor that prioritizes immediate rewards over future rewards. The expectation is taken over a trajectory of states and actions generated by the agent’s interactions with the environment. The value of a state s under a policy π is given by the state-value function $V^{\pi }(s)=E[R_{t}|s_{t}=s]$ which represents the expected return when starting from state s and following policy π. Similarly, the action-value function $Q^{\pi }(s,a)$ is $Q^{\pi }(s,a)=E[R_{t}|s_{t}=s,a_{t}=a]$, and quantifies the expected return when taking action a in state s and subsequently following policy π.

A key challenge in RL is estimating the value function especially in complex or continuous state-action spaces, such as the ones in agent-based modeling. Directly enumerating all possible states becomes infeasible, requiring the use of function approximation to estimate the corresponding value functions. Linear smoothers can approximate the action-value function Q(s,a), where s represents the current state and a the action. The estimator for Q(s,a) is derived by adapting (1) to approximate rewards (or discounted returns):

(4) $$\hat{Q}(s,a)=\sum_{\;j=1}^{n}w((s,a),(s_{j},a_{j}))\cdot R_{j},$$

where $w((s,a),(s_{j},a_{j}))$ are weights measuring the similarity between the current state-action pair (s,a) and past instances $\left(s_{j},a_{j}\right)$, and $R_{j}$ is the observed reward associated with the j-th instance. In multi-step sequential decision-making, we use the return, defined as the discounted sum of rewards accumulated over a sequence of steps. The weights, as in SL, are determined using a similarity function (e.g., a kernel) to ensure the estimation is localized and data-driven. As mentioned, the estimator in (Equation 4) minimizes the mean squared error between predicted and true values of the value function. By using the discounted return instead of the immediate reward, this approach implicitly performs Q-learning with function approximation.

By having an estimation of the value function, an agent can use a policy function to make informed decisions. A policy specifies the agent’s strategy for selecting actions in the state it is in. One common function for this purpose is the Boltzmann function:

(5) $$P(a|s)=\frac{e^{\beta Q(s,a)}}{\sum_{a^{\prime }}\;e^{\beta Q(s,a^{\prime })}}$$

where β is the exploration-exploitation trade-off parameter, balancing the choice between trying new actions (exploration) and leveraging known rewards (exploitation). Lower values of β encourage exploration by assigning nearly equal probabilities to all actions, while higher values promote exploitation by favoring actions with higher estimated rewards.

### 3.3 Cognitive instance-based learning

Now that we have established the statistical learning foundations of our framework, we describe how these principles are implemented in the CogIBL model. The CogIBL model is based on the idea that decisions and behaviors have subjective utility (or value), such as satisfaction or preference. When a behavior occurs in a situation and produces an outcome, it is associated with a subjective assessment of its value. Following ACT-R theory, these experiential associations are stored in **declarative memory** as experiential records (chunks) of decision-making situations, behaviors, outcomes, and their values. Over time, this repository of experiences forms the basis for implicit and explicit knowledge about decision-making (72–74). It is assumed that when individuals are faced with decisions, they draw from these stored experiences, retrieving memories that align with current cues to evaluate alternatives and decide on actions. This relies on ACT-R’s memory **retrieval** and **blending** mechanisms. Retrieval uses situation cues to recall past instances based on their **recency**, **frequency** and **similarity** to the current situation. Blending aggregates and generalizes across activated memories. By leveraging instance-based knowledge the model is able to estimate expectations of potential outcomes based on past similar situations.

A typical learning mechanism of an RL agent is Q-Learning (75), which updates the Q-values using the following Equation 6:

(6) $$Q(s,a)=Q(s,a)+\alpha \left(R(s,a)+\gamma \max_{a^{\prime }\in A}Q(s^{\prime },a^{\prime })-Q(s,a)\right)$$

where α represents the learning rate, γ is a discount factor for future returns, and R(s,a) is the reward function. Here, $s^{\prime }$ denotes the next state resulting from taking action a in state s, and $a^{\prime }\in A$ represents all possible actions in the next state $s^{\prime }$. The term $\max_{a^{\prime }\in A}Q(s^{\prime },a^{\prime })$ captures the maximum estimated future reward obtainable from the next state $s^{\prime }$. However, due to the continuous nature of epidemiological simulations, enumerating all possible states becomes infeasible. To address this challenge, we employ CogIBL’s estimation capabilities to approximate the action value function. This involves formulating the problem as an RLFA task, where the estimation from blending process minimizes the mean squared error between received rewards and estimated rewards, as described in Section 3.2.2.

**Figure 2:**
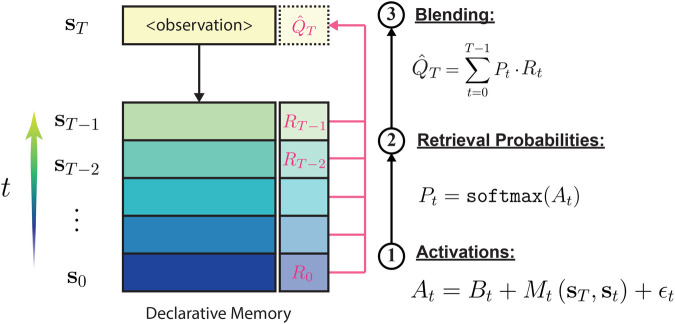
An overview of the CogIBL processes. CogIBL theory argues that implicit expertise is gained through the accumulation and recognition of previously experienced events. Events are stored in the Declarative Memory and are retrieved, weighted accordingly, in order to generate the model’s response.

In Figure 2 we describe in detail the computations that take place in the CogIBL model. The model approximates the utility for actions related to masking in three main steps:

1. **Activations Computation:** Each stored prior experience has an activation $A_{t}$ indicating its relevance to the current situation. Activations, reflect the cognitive mechanism of memory accessibility, modeling how prior usage and contextual relevance influence information retrieval from the declarative memory. This depends on two components, a temporal and a contextual one:
  a. The *Base-level activation* is the component of a memory chunk’s activation that reflects how frequently and recently that chunk has been used or retrieved. It is defined as (Equation 7):
    (7) $$B_{j}\;=\;\ln\left(\sum_{i=1}^{n}(t-t_{i}{)}^{-d}\right)$$
    where n is the number of past retrievals of chunk j, t is the current time (time of the retrieval attempt), $t_{i}$ is the time of the i-th previous retrieval of this chunk, and d is the decay parameter. Within ACT-R’s cognitive architecture, each chunk of knowledge accumulates “base-level activation” from previous retrievals. This accumulation decays over time, so chunks that were frequently or recently accessed are more likely to be retrieved again quickly.
  b. The *Matching Score* $M_{t}(s_{T},s_{t})$, measures the contextual similarity between the current state $s_{T}$ and the stored state $s_{t})$ based on a distance metric (e.g., cosine, Euclidean distance etc).
  The activation is a real-valued combination of these components with stochastic noise $\epsilon_{t}$ added, modeling stochastic memory recall. In our implementation, we set $B_{t}=0$ and $\epsilon_{t}=0$ to solely leverage the current context without historical biases or randomness. It is worth noting that the Matching Score can become more expressive by penalizing mismatches during the matching process or by using scaling factors for each component depending on the hypothesis being tested.
2. **Retrieval Probabilities:** Activations are normalized using the softmax function, producing probabilities that weigh past instances in the blending equation. These probabilities reflect the stochastic nature of memory retrieval, representing the likelihood of accessing specific information based on its activation level.
3. **Blending:** Decision output is the weighted average of past decisions $y_{t}$, weighted by their relevance to the current situation via retrieval probabilities. This outcome minimizes directly the mean squared error between model’s estimation and observed output. The process reflects the cognitive mechanism of generalization and interpolation, modeling how the mind combines multiple pieces of information to produce a composite response when exact matches are unavailable.

This approach conceptually aligns with Deep Q-Learning (34), where action values are estimated by a parametric neural network that approximates the Q value function. However, our framework alternatively leverages the non-parametric, instance-based regression native to our cognitive architecture. This enables cognitively-plausible RL within the agent-based modeling simulation while preserving cognitive interpretation of the emerging behaviors. Unlike parametric models, which explicitly assume a specific (e.g., linear or non-linear) relationship between global and local information, our non-parametric approach makes no such assumptions, allowing for greater flexibility in capturing complex interactions among state features. Moreover, our model does not require a dedicated training phase; it can generate estimations with just a few instances, either pre-defined or acquired through experience.

## 4 Epidemiological case study

In this section, we demonstrate our framework with a case study on masking behavior during the COVID-19 pandemic. We develop a utility-based model where agents make decisions about mask-wearing based on balancing competing preferences. Each agent receives inputs about the global pandemic status, such as infection rates, and the local status through the proportion of infected individuals in their neighborhood. Decisions are driven by a utility function integrating factors such as conforming to neighbors’ behaviors, discomfort from extended mask usage, and personal infection risk tolerance. By adjusting only the utility parameters (keeping all other parameters fixed for consistency and easier interpretation), and embedding agents in different social network topologies, we can model how various motivations shape behavioral patterns over time. Using the instance-based learning properties of the cognitive architecture, agents learn optimal behaviors by drawing on memories of past outcomes. These simulations reveal how population-level infection dynamics emerge from individual decisions influenced by varying motivations and social structures. Our framework enables testing of behavioral mechanisms driving protective measures and evaluation of policies to promote public health compliance during pandemics.

### 4.1 Agent-based modeling in epidemiology

We employ an agent-based SEIR (Susceptible, Exposed, Infectious, Recovered) epidemiological model, where agents transition through SEIR states. The infectious period includes pre-symptomatic, symptomatic, and asymptomatic phases, with geometrically distributed durations specified in Table 1. The model runs on daily timesteps, with infection spreading between neighboring agents on a transmission network. After recovery, agents maintain immunity for 75 days before becoming susceptible again. Most of these disease parameters represent characteristics typical of potential pandemic pathogens and are similar to early COVID-19 variants. We chose low immunity duration, a high reproduction number, and a high masking efficacy so that we could observe many waves of infection over a relatively short simulation interval and so that we could observe changes in epidemiological outcomes due to masking behavior.

**Table 1:** Epidemiological ABM parameters.

Variable	Value	Source
Disease state duration		
Exposed	2 days	Based on durations of early COVID-19 variants (76–78)
Presymptomatic infectious	3 days	Based on durations of early COVID-19 variants (76–78)
Symptomatic infectious	8 days	Based on durations of early COVID-19 variants (76–78)
Asymptomatic infectious	8 days	Based on durations of early COVID-19 variants (76–78)
Sterilizing immunity	75 days	Shorter than typical COVID-19 immunity periods so that we could observe many waves over a short time (79)
Transition probabilities		
Asymptomatic proportion	0.2	Based on asymptomatic proportion of early COVID-19 variants (80–82)
Disease transmission		
Basic reproduction number	5	Higher than typical COVID-19 reproduction number so that we could observe many waves over a short time (83, 84)
Random mixing proportion	20%	Accounts for low probability interactions
Initial exposed proportion	1%	Initial condition
Masking effectiveness	80%	Higher than typical masking effectiveness, so that we could observe changes in epidemiological outcomes due to masking behavior (85, 86)

The network consists of nodes (agents) and edges (contacts between agents), with edge weights representing daily transmission probabilities. The primary network in our study is a synthetic socio-centric graph of Portland, Oregon developed by the Network Dynamics and Simulation Science Lab at Virginia Tech (87). This dataset contains is a representation of daily social interactions in an urban setting and has previously been used to model infectious disease transmission dynamics (88). Due to computational constraints, we reduced the network to approximately 10,000 individuals using an iterative clustering method that preserves key structural properties, such as degree distributions and demographic mixing matrices. Alternative networks, including random unweighted graphs and Barabási-Albert Scale-Free graphs, were generated to explore the impact of network topology on disease dynamics and learning processes (for more details refer to the Supplementary Material).

We calibrated network transmission by scaling edge weights to achieve a target basic reproduction number ($R_{0}$). Each edge between susceptible and infectious agents has a weight-based probability of transmission, with masking reducing both infection and transmission risks. Social network data may not include low probability contacts—such as the small chance that a single person infects each other person in a crowded public space like a concert venue or supermarket. To capture these interactions, we allocate 20% of the $R_{0}$ to random mixing. For random mixing, we calculate the expected number of infections based on the $R_{0}$, number of infected people, number of susceptible people, and aggregate mask wearing behavior. We then randomly assign these expected infections to susceptible individuals throughout the network. This hybrid approach combining network and random transmission captures both structured social contacts and stochastic community transmission.

### 4.2 CogIBL implementation

**Figure 3:**
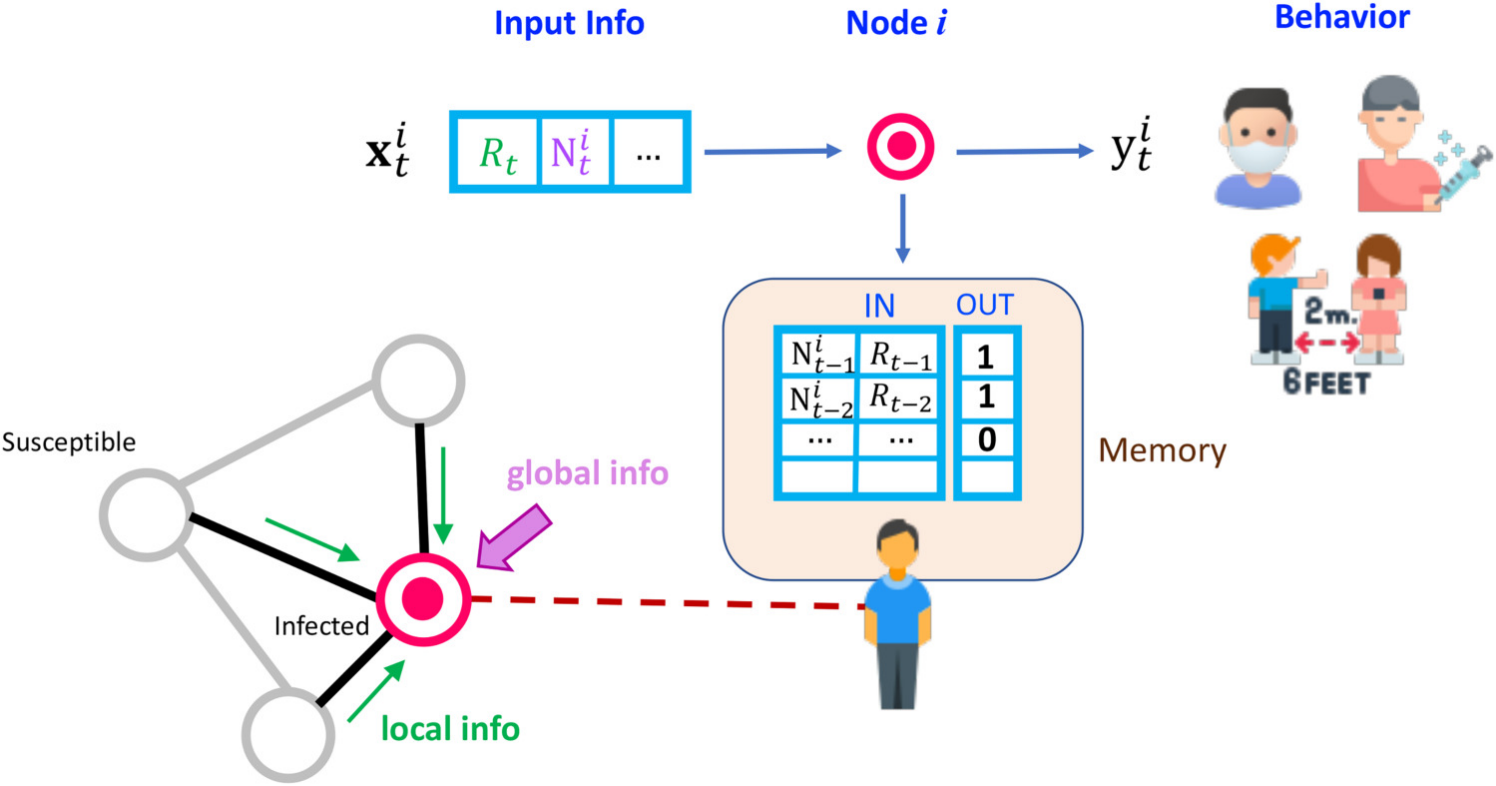
Example of agent’s decision-making in epidemiological ABM simulation.

We implement the CogIBL framework outlined in Section 3.3 as the core decision-making mechanism for our agents in the mask-wearing problem. An illustration is depicted in Figure 3 and a detailed mapping of the framework concepts to their implementation, including states, weights, and outputs, is provided in Table 2. At every timestep t, agents perceive the current state of the system $s_{T}=(M_{\text{local}},I_{\text{local}},I_{\text{global}})$ of the proportions of masked $M_{\text{local}}$ and infected neighbors $I_{\text{local}}$, and the global proportion of infected individuals $I_{\text{global}}$, combining local and global information from the disease transmission network. The agent then compares current state $s_{T}$ with previously stored instances $s_{t}$ using the similarity function $M(s_{T},s_{t})$ defined in Table 2. Based on this similarity, activations are computed and normalized to derive retrieval probabilities, which are then used to blend prior outcomes and estimate the action-value function Q(s,a), which quantifies how preferable it is for the agent to (un)mask given the current state of the pandemic. After an action, the agent receives a reward based on criteria described in detail in Section 4.4. In our implementation, we pre-populate all agents’ memories with the true utility values for the extreme cases (boundaries) of each state variable, assuming that humans operate within similar known bounded ranges. This initialization constrains agents’ interpolated utility estimations and resulting actions to remain within reasonable bounds, even at the start of the simulation.

**Table 2:** Mapping of concepts from the proposed framework to the actual implementation of the mask-wearing decision-making problem.

Framework concept	Implementation details	Explanation/notes
Memory instances	Stored experiential records (chunks)	Represent decision-making situations, behaviors, and outcomes.
Inputs s	$s_{t}=(M_{\text{loc}},I_{\text{loc}},I_{\text{global}})$	Combines local mask-wearing rates, local infections, and global infections.
Weights w(.)	$P=\text{softmax}(A_{t})$	Probabilities derived from activations $A_{t}$.
Outcomes $R_{j}$	$R(s_{t},a_{t})$ (Equation 8)	Reward function evaluating discomfort, conformity, and risk.
Activation $A_{t}$	$M(s_{T},s_{t})$, $B_{t}=\epsilon_{t}=0$	Similarity of memory instances.
Decision policy π	P(a|s)=softmax(βQ(s,a))	Maps state-action pairs to probability of masking.
Blending	$Q(s,a)=\sum_{j}P_{j}\cdot R_{j}(s,a)$	Weighted average of past rewards.

### 4.3 Decision making

We hypothesize that agents do not extensively plan for the longer-term future when deciding whether to wear a mask. Instead, they assess criteria relevant to the present moment, based on the local and global pandemic information they receive. To capture this short-term reward optimization, we assume each choice as an independent trial and set the reward discount factor γ=0 to make rewards dependent solely on the immediate state rather than future states. Each agent follows the policy defined in Equation 5. For our purposes it was set to β=5 so the agents are leaning towards exploitation. We allow agents to change their policies every 7 days.

### 4.4 Reward function

At every step, the agents receive a scalar reward value as feedback for their action. We assume that mask-wearing is a behavior that depends on a multitude of factors which have to do with the internal reward system of each individual rather than external factors. For this, we define an intrinsic reward function that we provide to agents based on evaluating their current state and actions regarding mask-wearing decisions. This scalar utility results from the weighted sum of three key reward components:

(8) $$R(s,a)=-w_{1}\cdot \text{DP}+w_{2}\cdot \text{CR}+w_{3}\cdot \text{RR}$$

The reward components are defined as follows:

• **Discomfort penalty (DP):** This penalty represents the relative agent’s discomfort with mask-wearing. DP is defined as DP=−a
• **Conformity reward (CR):** This reward promotes an agent’s conformity to the mask-wearing behaviors of neighboring agents. CR is defined as $\text{CR}=1-|a-M_{\text{local}}|$ where $M_{\text{local}}$ is the proportion of masked neighbors.
• **Risk reduction reward (RR):** This reward promotes an agent’s perception of infection risk reduction from wearing masks. RR is defined as $\text{RR}=a(1-mf)(c\cdot I_{\text{local}}+(1-c\cdot I_{\text{global}}))$, where mf is the masking factor indicating the propensity of virus transmission when an agent wears a mask (mf=0 means 0 probability of virus transmission), c a constant that represents how much an agent values infections in its neighborhood, and $I_{\text{local}}$ and $I_{\text{global}}$ the proportion of infections in agent’s neighborhood and the whole network respectively.

By tuning the relative weights of these utility factors, we can elicit varying motivational drivers that produce emergent mask-wearing behaviors. The agents learn probabilistic mask-wearing policies to maximize their utility over time using the rewards from their decisions in the changing pandemic environment.

## 5 Results

We analyze outcomes under different configurations of the conformity, discomfort, and risk reduction weights composing the mask-wearing utility function. Experiments compare two underlying social network topologies over which the disease simulation occurs. For each parameter combination and network, simulations are initialized identically and run until conclusion of the pandemic wave.

### 5.1 Modeling behavior

**Figure 4:**
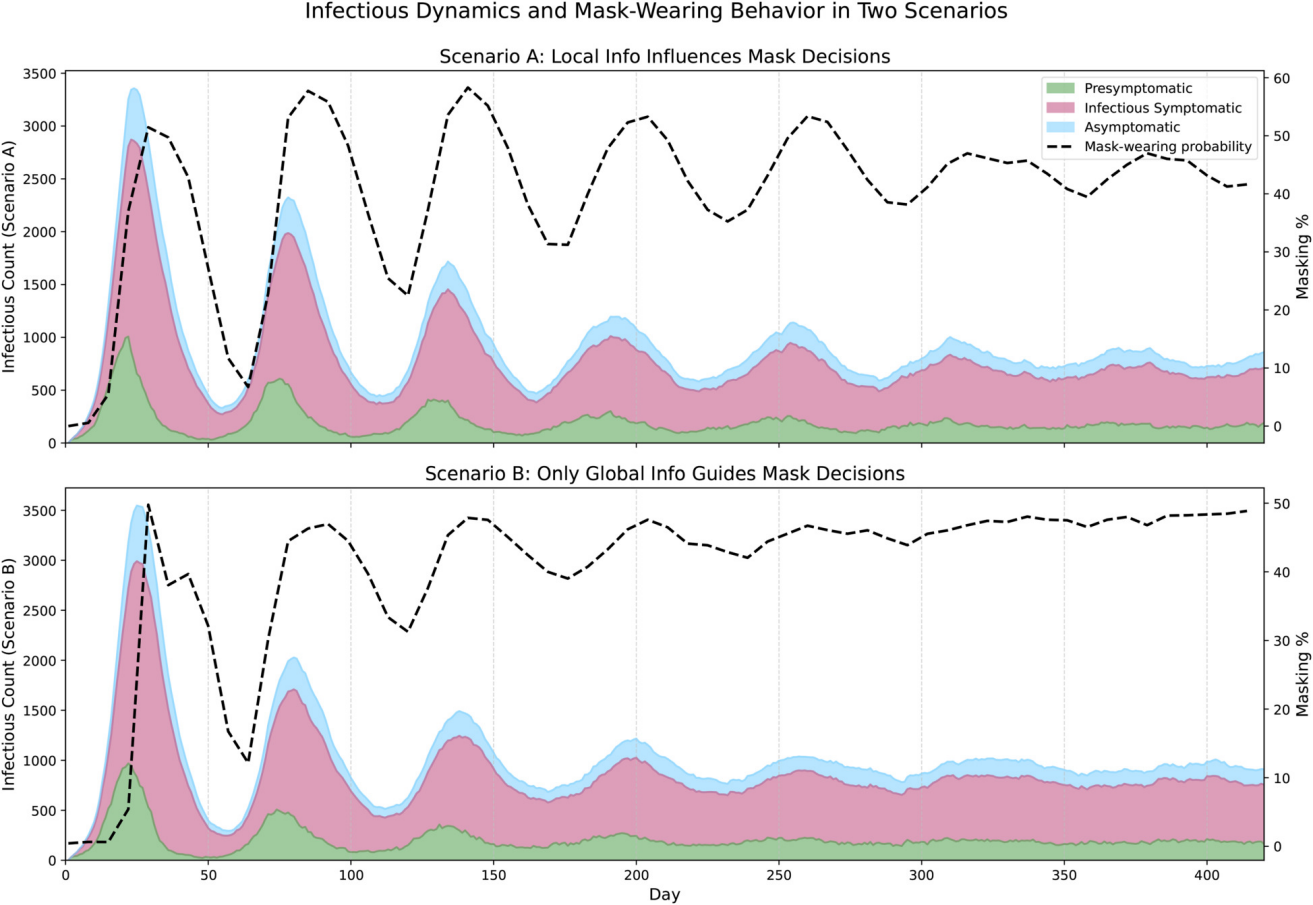
Epidemic evolution in the Portland network under two behavioral scenarios. The **top panel** shows the case where agents respond to both *local and global information* (c=0.8, $w_{1}=w_{2}=0.5$, $w_{3}=7.5$). The **bottom panel** shows the dynamics when agents base mask-wearing decisions only on *global infection information* (c=0.0, $w_{1}=0.5$, $w_{2}=0.0$, $w_{3}=7.5$). In each panel, the stacked area plot shows the number of agents in each infectious state (Presymptomatic, Infectious Symptomatic, Asymptomatic), while the dashed black line represents population-wide mask-wearing probability over time.

Figure 4 compares epidemic dynamics and masking behavior in the Portland network under two behavioral scenarios. The area plot shows the number of nodes in infectious states over time, and the proportion of mask-wearing is shown as a line plot on a secondary axis. The **top panel** shows the case where agents incorporate both *local and global information* in their decision-making. Here, masking behavior fluctuates more frequently, as individuals respond to varying local infection levels in their neighborhoods. These asynchronous behaviors lead to more irregular epidemic waves. In contrast, the **bottom panel** shows the evolution of the pandemic when agents respond exclusively to *global infection information*. In this scenario, masking behavior is highly synchronized across the network: once the global signal crosses a threshold, agents tend to increase masking in unison. This results in higher and more sustained masking levels overall, producing smoother epidemic waves.

**Figure 5:**
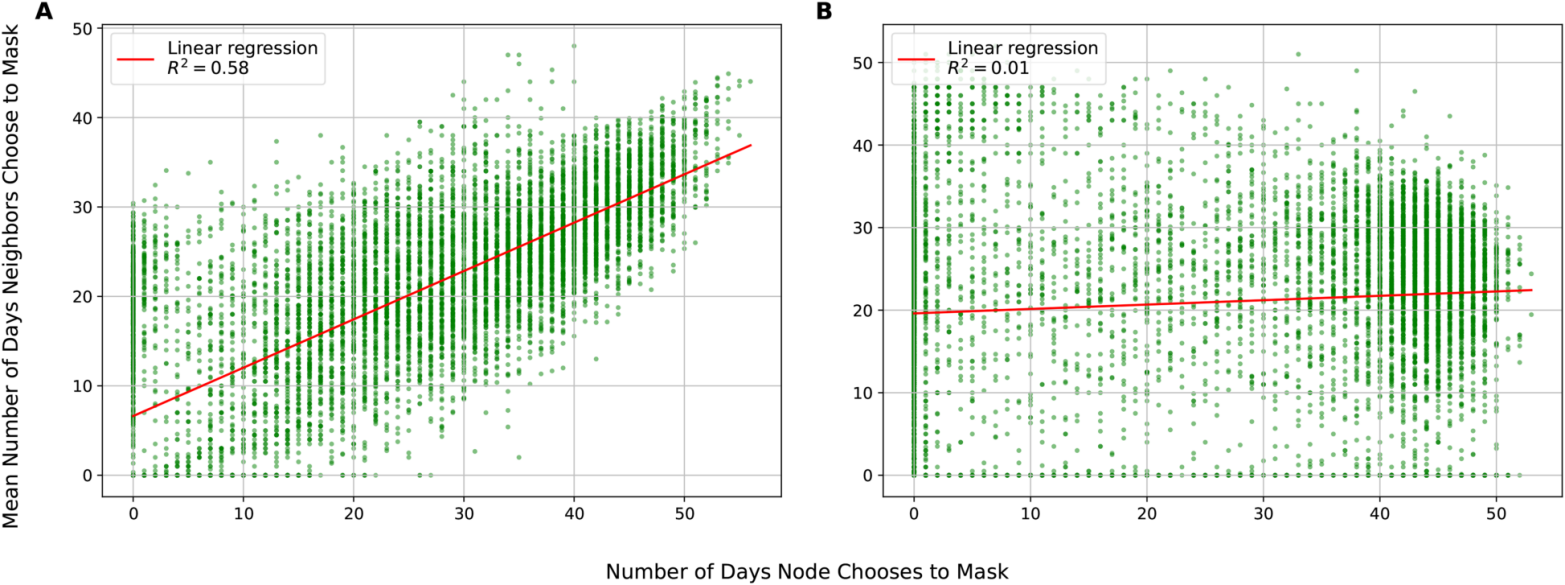
Assortativity in Portland network. (**A**) Local infection parameter c=0.8, $w_{1}=w_{2}=0.5$ and $w_{3}=7.5$. (**B**) Local infection parameter c=0.0, $w_{1}=0.5$, similarity parameter $w_{2}=0.0$ and $w_{3}=7.5$.

Figure 5A shows masking assortativity plots using the Portland network for two conditions: the base case in which individuals have access to local and global information and a scenario where they can only observe the global state. These plots show how the masking behavior of a node’s neighbors changes as a function of that node’s behavior across the entire duration of the simulation. The upward-sloping line for the local information condition shows that masking is assortative: that masking behavior clusters together with some regions of the network masking and other regions not masking. The gradient of the line is 0.54, implying for each day an agent spent masking, their neighbors will, on average, spend 0.54 days masking. The Pearson correlation coefficient is 0.76, indicating that the vast majority of the variation in individual masking behavior is captured by the behavior of neighbors (and vice-versa). In contrast, under the global only condition (Figure 5B), there is weak disassortativity, with gradient of 0.05 and a Pearson correlation of 0.09, suggesting that agents mask largely independently of their neighbors. This difference in behavioral coordination is reflected in epidemic outcomes: The local+global condition yields a Final Epidemic Size (FES) of 36.1%, a peak incidence of 523, and a time to peak of 19 days. Under the global-only condition, the FES rises to 43.3%, peak incidence reaches 544, and the peak occurs earlier at 18 days. Additional simulation runs with varying parameter settings and their corresponding outcomes (FES, peak incidence, and time to peak) are reported in the Supplementary Material.

Coordination of masking behavior is real-world phenomena: some communities have high levels of masking while others have low levels of masking, even when facing similar pandemic conditions. There was large variation in masking adoption across US states, and people rural areas tended to wear fewer masks than those in urban areas (89). Differences in the adoption of preventative measures can potentially lead to differences in outcomes: such as the high case rates observed in rural areas (relative to urban areas) (90). Agent-based network approaches like the one we use in this paper are able to capture these local variations, whereas population based approaches like system dynamic models using differential equations, cannot.

## 6 Advantages and extentions of CogIBL in epidemiological models with human behavior

CogIBL was directly tailored for the specific application of modeling mask-wearing behavior, but its versatility makes it applicable to a wide range of scenarios. In this section, we outline its key advantages and potential extensions for future work:

**Cognitive salience:** Similar to the concept of gradient-based salience (91), we can define cognitive saliences (92). These saliences measure the sensitivity of the value function to variations in input state features (e.g., proportion of infected neighboring nodes). The method provides an interpretation of agent’s decisions by identifying the most influential inputs driving behavior.

**Learning and adaptability capabilities:** As a non-parametric instance-based learning model, CogIBL does not require a typical training phase like parametric models do, reducing the computational overhead during simulations. Instead, it keeps the “training data” within its memory repository, allowing it to adapt dynamically to new situations. This is particularly useful in implementing cognitively-plausible algorithms for decision making as the model acquires experience and learns from it by interacting in real-time with the other agents in the agent-based simulation. Learning relies on comparing new experiences to the agent’s memory rather than propagating gradients through layers of predefined parameters, as its typical with neural networks. This mirrors human-like rapid decision adjustment based on accrued observations.

**Scalability:** To accommodate large datasets, CogIBL computations can be vectorized and parallelized, supported by techniques such as approximate^2^ k-nearest neighbors (93–95) for efficient scalability.

**Language capabilities:** Park et al. (96) implemented structurally similar memory and retrieval mechanisms to accommodate generative agents (GA) with language capabilities using Large Language Models (LLMs). Both GAs and CogIBL store past experiences as memory instances and retrieve relevant information based on similarity and context. This similarity extends to language capabilities, as CogIBL can incorporate components for natural language reasoning and be integrated with LLMs, as discussed in (97). This integration enables agents to be equipped with realistic behavioral profiles and simulate human-like cognition, decision-making and linguistic interactions. Recent work demonstrated simulations involving up to a million agents (98), where natural language serves as a medium for reasoning, planning, and interaction with other agents, allowing large-scale modeling of human behavior, such as misinformation propagation or adaptive responses to social phenomena. Williams et al. (99) demonstrated the use of GA variations in epidemiological networks and agent-based simulations.

**Data-driven processes:** CogIBL, as a statistical learning model, enables high-fidelity simulation of human behavior by incorporating empirical data from survey responses (100), social media or other sources, directly into agents’ memory structures. This allows agents to begin simulations with realistic initial experience based on real-world observations rather than abstract rules or assumptions.

**Non-linearity:** Linear smoothers assume a linear relationship between predicted outputs and training outputs, with weights $w(x,x^{\prime })$ determined solely by input similarity. In contrast, bilateral filters (101) introduce non-linearity by making the weights dependent not only on the input features but also on the output values (e.g., $w(x,x^{\prime },y,y^{\prime })$), resulting in a non-linear relationship between predicted and training outputs. This non-linear property is particularly relevant in epidemiological settings where the same decision might have drastically different impacts under varying circumstances. For instance, while masking during an influenza outbreak might have minimal effect on an agent’s fitness, the same behavior during a Spanish flu outbreak could significantly improve outcomes. From a CogIBL perspective, even if the experiential cues (e.g., infected neighbors) are identical, the action’s value can vary dramatically depending on the severity of the disease (e.g., mild illness vs. severe sickness). This ability to account for such non-linear relationships enhances the realism and flexibility of the framework in complex decision-making scenarios.

**Collective decision-making:** The RL capabilities can be extended to multi-agent reinforcement learning (MARL) to account for both individual incentives and community interests, or balance personal and group preferences. For example, in an agent-based simulation, an individual agent may prioritize personal incentives, but during working hours at a care facility, it can adopt safety protocols to protect the well-being of the community. These extensions align with the ‘utility calculus’ concept, where agents are seen as utility maximizers, and with social affiliation concepts, which integrate interpersonal and collective utilities and individuals adopt the goals and needs of others to maintain relationships (102, 103). This approach resonates with group and multi-level selection theories in evolutionary game theory, where cooperation within a group enhances the overall fitness of the community, even if it may not maximize individual fitness (104–106). The properties of CogIBL can be extended to incorporate alternative smoothing approaches inspired by linear filters like the mean filter (107), and nonlinear ones such as the bilateral and the non-local (108) filters. For example, in scenarios where individuals lack relevant experiences and are uncertain about decisions, the blending mechanism in Section 3.3 can be modified to allow agents to adopt the average behavior of their peers (similar to a mean filter) or weigh actions based on similarity to their context or role (analogous to bilateral filters). By enabling decisions to depend on community dynamics rather than solely on past experiences, CogIBL provides the flexibility to model socially influenced decision-making, where behaviors are shaped by neighborhood or group interactions.

## 7 Discussion

In this work, we introduce a novel computational framework that integrates machine learning and cognitive modeling into agent-based simulations. Unlike parametric methods, the proposed approach leverages the IBL capabilities of the ACT-R architecture to approximate utility functions without requiring extensive training, enabling agents to adapt in real time to changing conditions in a cognitively plausible manner. The core components of the framework simulate human-like cognitive processes by modeling decision-making, memory retrieval, and learning mechanisms inspired by psychological theories. The application of this framework to mask-wearing behavior during the COVID-19 pandemic highlights its ability to capture adaptive behaviors in epidemiological contexts, providing insights into the relationship between individual decisions and population-level dynamics.

Our simulation of adaptive mask-wearing behaviors across networks led to several findings. When individuals learn from the local information (neighbors’ masking behavior and infection rates), they develop assortative masking behavior, similar to patterns observed across the US in the COVID-19 pandemic. This variation in preventive actions across the network caused the disease to spread differentially in different parts of the network, effectively damping oscillations in the number of cases. In contrast, when individuals were only able to react to global infection rates, case oscillations persist unchecked, potentially overwhelming healthcare resources. These contrasting disease transmission regimes demonstrate how individual responses to local conditions can significantly alter macro-level disease dynamics, highlighting the importance of incorporating adaptive behavior in epidemiological models.

The use of the cognitive architecture provides multiple advantages for epidemiological modeling over conventional reinforcement learning. First, the instance-based approach rapidly adapts to new pandemic data without requiring extensive offline dataset training, enabling real-time responsiveness. Second, by incorporating ACT-R cognitive principles, the model’s mechanisms and behaviors can be interpreted through established psychological theory. Third, this framework efficiently scales to thousands of socially-interacting autonomous agents, capturing phenomena like shared identity formation and conformity pressures during crises. This scalability allows us to examine how individuals balance personal choices against group dynamics–a critical consideration for developing context-sensitive public health policies. These capabilities make our framework suitable for creating interpretable, scalable simulations of human decision-making in epidemiological contexts.

To our extent of knowledge, this work, is among the first to explore how adaptive mask-wearing behavior and social networks shape the dynamics of a pandemic like COVID-19, and there are several limitations. First, we only explore mask wearing behavior. Future models could explore how short-term masking decisions impact longer-term measures like vaccination, or population-level policies like social-distancing. Second, we rely on on synthetic networks, which might not capture all the structural features relevant to COVID-19. Further work could look at cases where the percolation of behaviors (e.g., mask-wearing) and disease occur on different networks, or integrate real-world survey into network construction. Third, we do not allow for variation in risk perception and utility functions between individuals or over time. Future work could allow for variation in risk perceptions which are transmitted across contacts, or which are intrinsic to the individual, such as fatigue in complying with preventative measures. Finally, we do not calibrate our model to real-world data, limiting the applicability of our findings to policy.

In conclusion, we believe that our framework can unlock further applications of cognitively plausible machine learning methods in epidemiological simulations with high fidelity. By equipping agents with adaptive, interpretable decision-making capabilities grounded in psychological principles, the framework enables the exploration of complex behavioral dynamics. This work provides a robust foundation for designing and evaluating public health interventions, contributing to the development of more effective, data-driven solutions to pressing epidemiological challenges.

## Data Availability

The original contributions presented in the study are included in the article/**Supplementary Material**, further inquiries can be directed to the corresponding author/s.

## Footnotes

^1^Non-parametric in this context means that linear smoothers do not assume a fixed functional form for the relationship between inputs and outputs, instead deriving predictions directly from the data using weighted averages of nearby observations. In contrast, parametric models make strong assumptions of the functional form relating inputs and outputs (e.g., $\hat{\;f}(x_{0})=\beta^{T}x_{0}$ where β are coefficients estimated from the data.)

^2^Approximate means that for a given search, the neighbors returned are an estimate of the true k-nearest neighbors.
