# Supplementary material for: Cognitively-plausible reinforcement learning in epidemiological agent-based simulations
Source: Front Epidemiol. 2025 Jul 28;5:1563731. doi: 10.3389/fepid.2025.1563731 (PMC12336203; doi:10.3389/fepid.2025.1563731)
Supplement: Supplementary file 1 [file Datasheet1.pdf]

Supplementary Material

1 EPIDEMIOLOGICAL NETWORK

The dataset represents the entire city of Portland, over 1.6 million individuals, which is too large to simulate in a timely manner. To streamline this extensive network to around ten thousand individuals, we employed a method involving the selection of specific clusters from the original network. These clusters encompassed all internal edges connecting the sampled nodes and the peripheral edges or stubs. We proceeded on an iterative process to connect these stubs while aiming to preserve the original dataset's network degree distributions and demographic mixing matrices. This method essentially restructures connections within the network to maintain the fundamental structure of the original dataset. It involves two primary steps: first, reorganizing connections between nearby nodes, focusing on links between the inner and outer segments of the sampled network. Careful recalibration of these connections involves using calculated edge weights to establish meaningful associations between nodes linked to external ones. The second step entails rewiring connections between distant nodes, particularly those without commonalities with external nodes. This phase involves a thorough examination of the degree structures of these nodes, attempting to adjust connections while upholding the observed degree distributions in the original dataset. This iterative process aims to minimize disparities between the original and modified networks, ensuring that the distilled network maintains essential characteristics while providing a more manageable portrayal of Portland's intricate social network. This process yielded a network with 9,223 individuals and 102,623 edges. We generated alternative networks so that we could explore the impact of network structure on both disease percolation and learning processes. This included random unweighted and Barabási-Albert Scale-Free graphs, which were sampled such that the total number of nodes and edges matched the Portland network.

2 PSEUDOCODE FOR AGENT DECISION-MAKING MODEL

Algorithm 1: Agent-Based Simulation with Reinforcement Learning Behavior

Input: Population parameters, simulation duration T, RL agent parameters
Output: Simulation trajectories, agent decision logs, infection states

```
Initialize simulation parameters;
Build network G from population data;
Instantiate CogIBL agents at each node in G
for decision step t = 1 to T do
    for agent i in G do
        Observe infection state s_i (local + global);
        Select action a_i using agent's policy;          /* agent's decision making */
        Apply behavior based on a_i (e.g., update transmission factor);
        Record s_i, a_i, utility estimates, and action probabilities
    Run epidemic simulation for d days;
    Update network G with new infection and behavior states;
    for each agent i in G do
        Compute reward r_i = R(s_i, a_i);
        Update agent using (s_i, a_i, r_i);                /* learning */
Output results and save simulation history;
```

3 SUMMARY OF SIMULATION SCENARIOS AND OUTCOMES

Table S1. Summary of simulation results for different parameter configurations.

c (Local Info)	w_1 (Discomfort)	w_2 (Conformity)	w_3 (Risk)	FES	Peak	TTP
1.0	0.5	0.5	7.5	0.3607	523	19
0.8	0.5	0.5	7.5	0.3605	546	17
0.4	0.5	0.5	7.5	0.3823	535	17
0.0	0.5	0.5	7.5	0.7455	566	17
0.0	0.5	0.0	7.5	0.4326	544	18
0.1	0.5	1.0	7.5	0.7533	566	17
1.0	0.5	0.0	7.5	0.3579	528	19
0.8	0.5	0.0	0.0	0.7376	544	18
0.8	0.5	1.0	0.0	0.7512	566	17
1.0	0.5	1.0	7.5	0.4560	545	17

Notes: FES = Final Epidemic Size; Peak = Peak Incidence; TTP = Time to Peak. All simulations use $\beta = 10$, $\gamma = 0$, and softmax temperature $\tau = 0.1$.

4 INTERPLAY OF HUMAN BEHAVIOR AND EPIDEMIOLOGICAL DYNAMICS

Collective masking behavior and infection prevalence evolve jointly over time under different behavioral assumptions. In this section, we report the phase-space trajectories of masking probability versus infection rate, for the two main scenarios examined, to demonstrate how these coupled dynamics unfold.

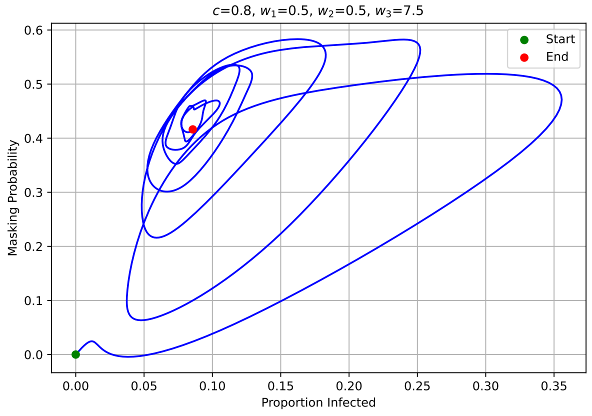

Figure S1. Phase portrait-like plot showing the relationship between infection prevalence and masking probability in the Portland network, when agents respond to both *local and global information*.

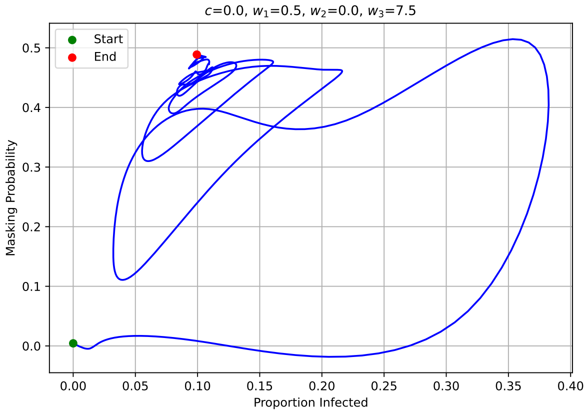

Figure S2. Phase portrait-like plot showing the smoothed trajectory of infection prevalence and masking probability in the Portland network when agents respond only to *global infection information*.
